# Soil water stress affects both cuticular wax content and cuticle-related gene expression in young saplings of maritime pine (*Pinus pinaster* Ait)

**DOI:** 10.1186/1471-2229-13-95

**Published:** 2013-07-01

**Authors:** Grégoire Le Provost, Frédéric Domergue, Céline Lalanne, Patricio Ramos Campos, Antoine Grosbois, Didier Bert, Céline Meredieu, Frédéric Danjon, Christophe Plomion, Jean-Marc Gion

**Affiliations:** 1 INRA, UMR 1202, BIOGECO, F-33610, Cestas, France; 2 Univ. Bordeaux, BIOGECO, UMR 1202, F-33400, Talence, France; 3 Univ. Bordeaux, Laboratoire de Biogenèse Membranaire, UMR5200, F-33000, Bordeaux, France; 4 CNRS, Laboratoire de Biogenèse Membranaire, UMR5200, F-33000, Bordeaux, France; 5 Instituto Biología Vegetal y Biotecnología, Universidad de Talca, 2 Norte 685, Talca, Chile; 6 CIRAD, UMR AGAP, Campus de Baillarguet TA 10C, F-34398, Montpellier Cedex 5, France

**Keywords:** Cuticle biosynthesis, Drought, Edaphic stress, Field experiment, Gene expression, Maritime pine

## Abstract

**Background:**

The cuticle is a hydrophobic barrier located at the aerial surface of all terrestrial plants. Recent studies performed on model plants, such as *Arabidopsis thaliana*, have suggested that the cuticle may be involved in drought stress adaptation, preventing non-stomatal water loss. Although forest trees will face more intense drought stresses (in duration and intensity) with global warming, very few studies on the role of the cuticle in drought stress adaptation in these long-lived organisms have been so far reported.

**Results:**

This aspect was investigated in a conifer, maritime pine (*Pinus pinaster* Ait.), in a factorial design with two genetic units (two half-sib families with different growth rates) and two treatments (irrigated vs non-irrigated), in field conditions. Saplings were grown in an open-sided greenhouse and half were irrigated three times per week for two growing seasons. Needles were sampled three times per year for cuticular wax (composition and content) and transcriptome (of 11 genes involved in cuticle biosynthesis) analysis. Non-irrigated saplings (i) had a higher cuticular wax content than irrigated saplings and (ii) overexpressed most of the genes studied. Both these trends were more marked in the faster growing family.

**Conclusions:**

The higher cuticular wax content observed in the non-irrigated treatment associated with strong modifications in products from the decarbonylation pathway suggest that cuticular wax may be involved in drought stress adaptation in maritime pine. This study provides also a set of promising candidate genes for future forward genetic studies in conifers.

## Background

The cuticle is the outermost layer of the aerial parts of the plant. This protective coat is synthesized exclusively by epidermal cells and has two major constituents: cutin, a lipophilic polymer, and cuticular waxes, which are embedded in and cover the cutin. The cutin matrix consists mostly of inter-esterified 16- and 18-carbon atom fatty acids, whereas waxes are produced from very long-chain fatty acids (VLCFA, reviewed by [1,2]). VLCFA are synthesized by fatty elongase complexes located in the endoplasmic reticulum, from C16-C18 fatty-acid precursors synthesized *de novo* in the plastids [3]. The constituents of the wax layer are then synthesized from this VLCFA pool via two independent wax biosynthesis pathways in epidermal cells: (i) the acyl reduction pathway, generating primary alcohols and wax esters and (ii) the decarbonylation pathway, which produces aldehydes, alkanes, secondary alcohols and ketones (reviewed by [2,4-6]). The identification of 24 *Arabidopsis thaliana* mutants with modified cuticular wax composition (*eceriferum* or *cer* mutants; [7,8]) led to the molecular characterization of several proteins involved in cuticular wax biosynthesis and deposition (reviewed in [2]).

Cuticular waxes account for 20 to 60% of cuticle mass. The cuticle is involved in protection against UV-light, fungal and bacterial pathogens, the prevention of post-genital organ fusion and in plant-insect interactions [9]. However, cuticular waxes are principally involved in limiting non-stomatal water loss and therefore constitute a key adaptation in the evolution of land plant [6,10]. Indeed some authors [11] reported that cuticular wax compounds may increase resistance to water diffusion in an artificial membrane. Further evidence in support of this hypothesis has been provided by recent studies showing that plants exposed to drought stress have higher levels of cuticular wax deposition [3,12,13]. In Arabidopsis, a specific increase in alkane content was recently correlated with greater drought tolerance [14]. The overexpression of a transcription factor involved in cuticular wax regulation (i.e. SHINE) has also been shown to increase cuticular wax deposition and enhance drought tolerance in both *Arabidopsis thaliana*[15] and *Medicago sativa* L. [16].

With global climate change, forest trees will have to cope with more frequent droughts. These climatic changes will occur over a time scale of one to a few generations, a period too short for perennial species to migrate as a mean of coping with such stressful conditions. Improvements in our understanding of the plastic response (including that involving cuticle biosynthesis) in these long-lived organisms are therefore required. The molecular mechanisms underlying drought stress adaptation in trees have been studied essentially in poplar and pine. Most studies in pine have used a wide range of genomic approaches to study transcriptional dynamics in the aerial parts [17] and roots [18-20] of water-deprived seedlings. However, the molecular mechanisms involved in cuticle biosynthesis are unknown in these species. A few studies of forest and fruit trees have reported changes in cuticular wax composition during leaf development [21], and abiotic (i.e. ozone) stress [22]. However, the relationship between cuticular wax composition and drought stress response has never been investigated in these perennial plants.

The main objective of this study was to evaluate the role of cuticular waxes in the drought stress response in maritime pine (*Pinus pinaster* Ait.), the first conifer used for the reforestation of south-western Europe. We used a factorial field design with two sets of irrigation conditions (irrigated vs. non-irrigated) and two genetic units (families with different growth rates). Water stress was imposed by growing the saplings in an open-sided greenhouse and irrigating only half the saplings, with aerial sprinklers, over two growing seasons. Needles were then sampled three times per year, for each set of irrigation conditions and each genetic unit, for (i) characterization of epicuticular waxes by gas–liquid chromatography, and (ii) qPCR determination of the expression profile of 11 genes involved in cuticular wax biosynthesis. Particularly in the rapidly growing family, non-irrigated plants had more cuticular wax than irrigated plants and overexpressed most of the genes analyzed. This study suggests that the cuticle and cuticular waxes in particular, may be involved in the drought stress response of pines, and provides a first insight on its genetic variation.

## Results

### Plant material and drought stress application

**Figure 1:**
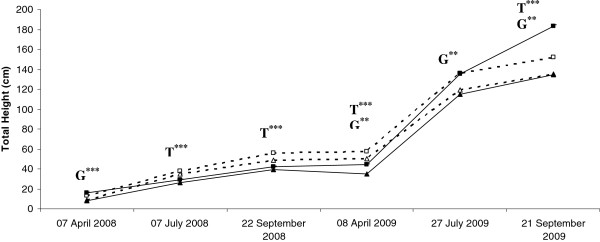
**Total height of the sapling over the two growing seasons.** Dotted and solid lines represent the non-irrigated and irrigated treatments, respectively. Triangles and squares represent the “V-” and “V+” families, respectively. Abbreviations are as follows: T: treatment effect, G: family effect. **P value < 0.001 and ***P value < 0.0001.

The main goal of this study was to investigate the role of the cuticle in the drought stress response of maritime pine saplings and its relationship to growth performance. Two maritime pine families (i.e. genetic units) with different growth rates were analyzed. A family with improved biomass production (V+) and the non-improved family (V-) were planted in February 2008, in a field factorial design. Drought stress was achieved by rainfall exclusion in a greenhouse tunnel divided in irrigated and non-irrigated zones. In each growing season (2008 and 2009), sampling was performed in early spring (April), early summer (July) and late summer (September), when drought stress was assumed to be the most intense. Saplings total height was monitored before each sampling point over the two growing season (Figure 1). In 2008, both families (“V+” and “V-”) presented a higher growth rate in non-irrigated treatment than in the irrigated treatment (Table 1 and Figure 1). In 2009 a higher growth rate was observed for the “V+” family in the non-irrigated treatment compared to the “V-” family in non-irrigated as well as in the irrigated treatments. In conclusion, it should be noticed that especially for the non-irrigated treatment, the “V+” family had a higher growth rate (Table 1 and Figure 1) in both growing seasons compared to the “V-” family (both in the non-irrigated or irrigated treatments), suggesting a better tolerance to drought stress in this family.

**Table 1:** **Timing of treatment** (**T**), **genetic** (**G**) **and interaction** (**TG**) **effects for variables analysed**

		**7 April 2008**	**7 July 2008**	**22 September 2008**	**8 April 2009**	**27 July 2009**	**21 September 2009**
**Total Height**	**T**	NS	S***	S***	S***	NS	S***
	**G**	S***	NS	NS	S**	S**	S**
	**TG**	NS	NS	NS	NS	NS	NS
**Predawn leaf water potential**	**T**	NS	S**	S***	NS	S***	S****
	**G**	NS	NS	NS	NS	NS	NS
	**TG**	NS	NS	NS	NS	NS	NS
**Water content**	**T**	na	NS	NS	S*	NS	NS
	**G**	na	NS	NS	NS	NS	NS
	**TG**	na	NS	NS	NS	NS	NS
**CW content**	**T**	na	NS	S**	S**	S***	S**
	**G**	na	NS	S**	NS	NS	NS
	**TG**	na	NS	S**	NS	NS	NS

Abbreviations: *T:* treatment effect, *G:* family effect, *TG:* interaction effect, *NS:* non significant, *S:* significant (*P value < 0.1, ** *P* value < 0.01, ***P value < 0.001 and ****P value < 0.0001), *na* not available.

### Effect of drought stress on leaf water potential

**Figure 2:**
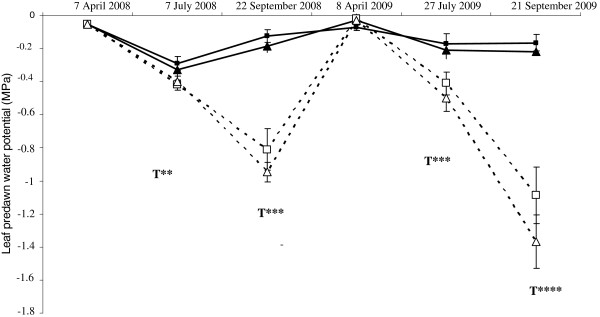
**Predawn leaf water potential over the two growing seasons studied (2008 and 2009).** Dotted and solid lines represent the non-irrigated and irrigated treatments, respectively. Triangles and squares represent the “V-” and “V+” families, respectively. Standard deviations were obtained from 6 independent measurements. Abbreviations: T: treatment effect. ** *P* value < 0.01, ***P value < 0.001 and ****P value < 0.0001.

Predawn leaf water potential was measured before each sampling, in both growing seasons, on needles from six individuals (Figure 2), to monitor the level of water deficit in the saplings. As expected, leaf predawn water potential declined steadily during both growing seasons for the non-irrigated treatment, but the stress was more intense in 2009 (−1.2 MPa) than in 2008 (−0.8 MPa). In young maritime pine saplings, a predawn leaf water potential below −1.1 MPa is considered to correspond to severe drought stress. Indeed, Delzon *et al*. [23] reported that maritime pine closes its stomata completely at predawn leaf water potentials below −1.8 MPa.

For the irrigated treatment, predawn leaf water potential was stable and close to zero, indicating that irrigation was sufficient to prevent evapotranspiration losses. In spring 2009, predawn leaf water potential was close to zero and similar for the two treatments (irrigated vs. non irrigated), due to an increase in the level of the water table (data not shown) during winter and the removal of the tarpaulin covering the greenhouse by Hurricane Klaus in January 2009. Statistical analysis showed that variation of predawn leaf water potential (Table 1) was mainly due to the treatment applied. No difference was observed between the two families.

### Drought stress affects cuticular waxes content

Cuticular waxes (CW) were extracted by chloroform dipping. The major peaks were identified by GC-MS (gas chromatography–mass spectrometry) and quantification was performed by GC-FID (gas chromatography-flame ionization detector), as previously described [14]. A representative GC profile is shown in Additional file 1: Figure S1. Nonacosan-10-ol, a C29 secondary alcohol with a hydroxyl group in position 10, was the main compound in all samples, accounting for 55 to 60% of total wax load. Fatty alcohols with even numbers of carbon atoms, ranging from C24 to C30, accounted for about 10-12% and C29 and C31 alkenes (i.e. monounsaturated alkanes) accounted for about 12-15% of total wax load. A compound accounting for 5 to 6% of total wax load (eluting shortly after 18 min) was tentatively identified as C29-hydroxy-alkene (C29AlkN-OH), and five unknown compounds systematically accounted for about 12% of total wax load. GC-MS analyses also revealed the presence of traces of saturated C29 alkane, 10-hydroxy C27 secondary alcohol, C33 alkene and C32 and C34 fatty alcohols (data not shown).

**Figure 3:**
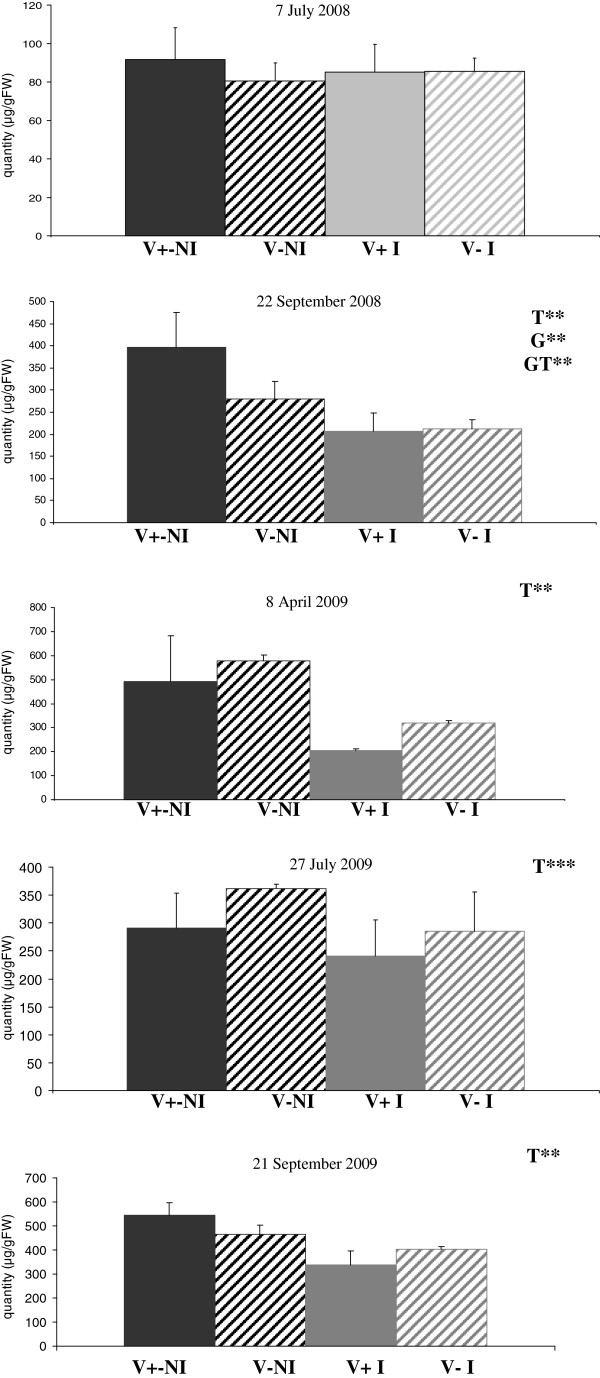
**Changes in cuticular wax content in 2008 and 2009.** Quantification was performed using three biological replicates. Abbreviations: V + NI: “V+” family, non-irrigated; V-NI: “V-” family, non-irrigated; V + I: “V+” family, irrigated; V-I: “V-” family, irrigated, T: treatment effect, G: family effect, GT: interaction effect, *P value < 0.1, ** *P* value < 0.01, ***P value < 0.001 and ****P value < 0.0001.

CW were then quantified on needles (same material used for transcriptome analysis, sampled from the last growth unit) harvested on 7 July 2008, 22 September 2008 (first growing season), 8 April 2009, 27 July 2009 and 21 September 2009 (second growing season). For each family and treatment, quantification was performed over three biological replicates (Figure 3). For all samplings, CW content was higher for the non-irrigated treatment. This pattern was particularly marked for needles harvested on 22 September 2008 (first growing season), 8 April 2009, 27 July 2009 and 21 September 2009 (second growing season) but was less pronounced for needles harvested on 7 July 2008 (first growing season). A statistical analysis was carried out to test for effects of treatment, family and their interaction (Table 1). In the first growing season, no significant differences were observed for needles sampled on 7 July, but needles harvested on 22 September displayed significant variations for all three effects considered. With the exception of the first sampling in the first growing season, the differences between the irrigated and non-irrigated treatments were confirmed, with a significant treatment effect identified and a higher CW content in drought-stressed plants. Except for needles harvested on 8 April 2009 where a significant treatment effect was identified, it should be noticed that the water content of the needles in all the experiment plots is not changed (Table 1) suggesting that the higher CW content observed in the non-irrigated treatment is probably due to the environmental conditions rather than variation in water content of the different samples analysed. A similar result on the needles water content was also obtained in Norway spruce submitted to drought stress [24].

Such a result could also be explained by differences in needles size between the two treatments. Nevertheless, if no phenotypic characterisation for this specific trait has been performed in our experiment, no differences were noted when collecting the samples.

### Search for cuticle biosynthesis-related genes

The following approach was used to determine whether genes involved in cuticle biosynthesis pathways were differentially regulated as a function of the factors investigated. First, the *Arabidopsis* database (http://www.arabidopsis.org) was used to recover the sequences of 136 genes involved in cuticle biosynthesis, as described by Costaglioli *et al*. [5]. These 136 *Arabidopsis* genes included 28 from the fatty acid elongation pathway, and 42 and 66 related to the wax and cutin biosynthesis pathways, respectively. Second, the Blast-X algorithm, (e-value cut-off < e-15) was used against the pine gene index available from the following URL: http://compbio.dfci.harvard.edu/tgi/cgi-bin/tgi/gimain.pl?gudb=pine. This led to the identification of 17 orthologous sequences (Additional file 2: Table S1): five from the fatty acid elongation biosynthesis pathway, seven from the wax biosynthesis pathways, three from the cuticle biosynthesis pathways (wax and/or cutin biosynthesis), one from the cutin biosynthesis pathway and one transcription factor involved in the regulation of cuticle biosynthesis. Six of these genes (*KCS1*, *FAR4*, *FAR6*, *WSD2*, *WSD3* and *CYP96*) displayed multibanding patterns on agarose gel electrophoresis after PCR amplification and were discarded from the analysis, and 11 (*SHINE1*, *ASAT1*, *WSD1*, *ECR*/*CER10*, *KCR1*, *CER6*/*KCS6*, *KCS4*, *LACS3*, *CER1*, *CER2*, *CER3*) were retained for quantitative gene expression analysis. PCR efficiencies were calculated from standard serial dilutions and ranged from 94% to 114%, these values being considered acceptable.

### Expression profiling during the first growing season

**Figure 4:**
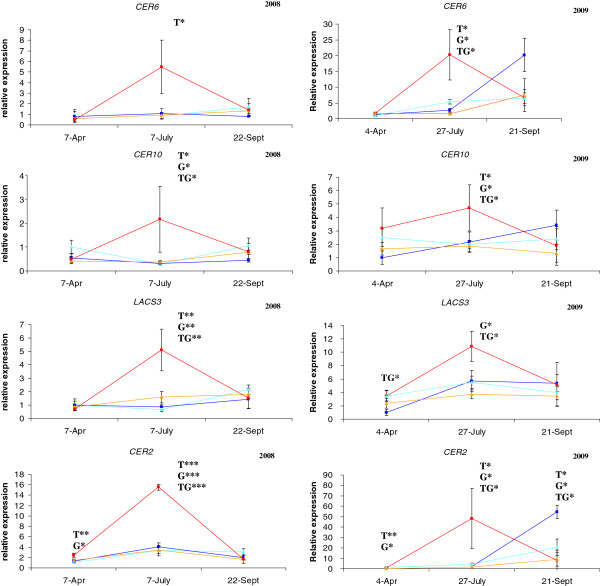
**Expression profile of the most relevant candidate genes identified over the two growing seasons.** Expression profiles for 2008 are on the left, whereas those for 2009 are on the right. Squares and red lines are used for the “V+” family for the non-irrigated treatment and triangles and orange lines are used for the “V-” family for the non-irrigated treatment. Squares and dark blue lines are used for the “V+” family for the irrigated treatment and triangles and light blue lines are used for the “V-” family for the irrigated treatment. Error bars represent the standard deviation (*N* = 3). Results of two-way ANOVA are also indicated above each sampling point. Abbreviations are as follows: T: treatment effect, G: family effect, TG: interaction effect. * *P* value < 0.01, **P value < 0.001 and ***P value < 0.0001.

Three of the 11 genes analyzed in the 2008 samples (Additional file 3: Figure S2, panels on the left, and Figure 4) displayed little variation in expression (*KCS4*, *ASAT1* and *WSD1*) and were considered not to be differentially expressed. The other eight genes belonged to four of the five main categories described in Additional file 2: Table S1 (fatty acid elongation: 3 genes; wax biosynthesis: 3 genes; cuticle biosynthesis: 1gene and transcription factor: 1 gene). Their expression profiles were similar, with expression levels maximal for the non-irrigated treatment in early summer (July), for family “V+”. This peak expression was generally followed by a decrease in transcript abundance. The expression profile of the most revelant candidate genes (CER6, CER10, CER2 and LACS3) is shown in Figure 4.

### Expression profiling during the second growing season

The same genes were quantified in 2009, with the exception of *KCS4* and *CER1*, which were analyzed only in 2008 (Additional file 3: Figure S2, panels on the right and Figure 4). Five of the nine genes had high standard deviations (*ASAT1*, *SHINE1*, CER3, KCR1 and *WDS1*) and were not considered to be differentially expressed. No significant effects were identified by ANOVA for *KCR1* and *CER3*, but these two genes appeared to be slightly overexpressed in July. As in the 2008 growing season, the other four genes (CER6, CER10, CER2 and LACS3) were most strongly expressed in July, in the “V+” family plants subjected to the non-irrigated treatment (Figure 4). Again, the expression peak was generally followed by a decrease in transcript accumulation.

## Discussion

### Stress applied and experimental design used

The molecular mechanisms involved in drought stress adaptation in perennial plants, including forest trees in particular, have mostly been studied in controlled experiments (reviewed by [25]). Most of these experiments were performed either in hydroponic conditions in growth chambers [17] or in pots in a greenhouse [19]. By contrast, this study was based on an original experimental design for studying the maritime pine drought stress response in field conditions. The strength of this experimental design lies in the analysis of the response to water deprivation in interaction with all other biotic and abiotic stresses faced by trees in natural stands. To ensure that water stress was the most important stress in the greenhouse, we monitored also the expression level of two genes (Lp3-3 in 2009 and PrAGP4, 2008 and 2009) known to be involved in the drought stress response of maritime pine [17,18]. A consistent expression profile was obtained (Additional file 3: Figure S2) suggesting that water stress was the dominating stress in our experiment. Moreover, pathogen attacks were visually monitored before each sampling point for all the saplings harvested. No pathogen attack was identified on the needles analyzed over both growing seasons. Finally the internal greenhouse temperature was also followed in 2008 and 2009. Except for needles harvested on 27 July 2009 where a very slightly higher temperature was registered (+0.9°C) no difference was identified. These results strengthen the idea that CW variations observed in our experimental design are mainly driven by the applied constraint (i.e. edaphic drought) rather than other abiotic stresses (heat temperature) or biotic stress (pathogens).

Even if a similar rainfall exclusion system has been used to study the ecophysiological response (changes in transpiration) of Holm oak (*Quercus ilex*) in non-irrigated environments [26] such experimental designs have rarely been used to study the molecular mechanism underlying the drought stress response in trees. Only one previous study investigated water stress-responsive proteins in a poplar field experiment [27]. However, no rainfall exclusion system was included in the design of this previous study to prevent water contamination during summer, resulting in only moderate water stress (−0.44 MPa) for the water-deficit conditions. In the present experiment, the non-irrigated saplings displayed a steady decrease in predawn leaf water potential in both growing seasons, consistent with the progressive establishment of water stress.

### Comparison of cuticle composition between maritime pine and Arabidopsis thaliana

The GC analyses provided the first demonstration that the cuticular wax of maritime pine needles consists predominantly of 10-hydroxy-C29 secondary alcohol (nonacosan-10-ol), which accounted for up to 60% of the total wax load, consistent with the results obtained for *Pinus halepensis*[28]. Overall, alkanes and their derivatives (alkenes and secondary alcohols) accounted for about 80% of the total wax load, with primary alcohols accounting for about 10%. These values are surprisingly similar to those reported for *Arabidopsis thaliana* stem waxes, in which alkanes (and their derivatives) and primary alcohols account for 75% and 13% (18% if those found in wax esters are included) of the total wax load [14]. The presence of these two classes of compounds in needle waxes suggests that, as in *Arabidopsis thaliana*, both the alcohol- and alkane-forming pathways function in maritime pine [29]. By analogy to the well described *Arabidopsis* pathways, it could be suggested that, in pine, fatty acids are first elongated and then either reduced to fatty alcohols or decarbonylated to yield alkanes. It remains to be determined whether the modifications of the alkane backbone (hydroxylation and double bonds) are introduced before or after decarbonylation.

### Impact of water deficit on the accumulation of transcripts of cuticle biosynthesis genes

In this study, the expression profile of 11 functional candidate genes involved in cuticular wax biosynthesis was determined, together with their regulation in response to soil water stress in two genetic units of maritime pine. This section focuses principally on the genes with different expression profiles in the two genetic units (i.e. showing a T x G effect), thereby highlighting potential candidate genes for drought stress adaptation in maritime pine. This interaction effect was generally detected in early summer, probably due to the choice of phenotypic trait analysed. Indeed, by late summer, the cuticle has already been synthesized by epidermal cells to protect the needles against non stomatal water loss, and there is therefore a decrease in transcript accumulation for the genes of this specific pathway. Furthermore, although no significant effect was detected for some of the genes analyzed in 2009 by ANOVA, with the exception of *SHINE1*, these genes (*i*.*e*. *KCR1*, *WSD1*, *CER3*; Additional file 3: Figure S2), had expression patterns very similar to those observed in 2008. This result may be first accounted for by the experimental design used in this study, in which only one parameter (*i*.*e*. water supply) was controlled precisely, resulting in analyses of the drought stress response in interaction with all the other stresses faced by forest trees in their natural environment. Second, it should also be borne in mind that the analyses were performed not only in different growing seasons, but also on different individuals (*i*.*e*. half sibs) presenting a certain degree of genetic variability. In addition to this within-family genetic variability, small differences in developmental stage between the needles analyzed may have affected the expression level observed. Such an effect has already been reported in a study of *Gerbera hyrida* in which young and old roots were compared [30].

Finally, an overexpression (generally, not significant) was observed for five genes (*CER6*, *CER10*, *KCR1*, *CER2* and *SHINE1*, Additional file 3: Figure S2, Figure 4) in late summer 2009, for family “V+” subjected to irrigation. This upregulation may be entirely due to the irrigation, which provides favourable environmental conditions for needle growth in late summer. This hypothesis is supported by the demonstration of wax deposition in the older leaves of another plant, growing barley [31].

The 11 candidate genes studied here can be classified into four groups according to the functions of the proteins they encode in cuticular wax biosynthesis and regulation: a transcription factor regulating cuticle biosynthesis (*SHINE1*), proteins involved in VLCFA biosynthesis (*CER6*/*KCS6*, *KCS4*, *KCR1*, *ECR*/*CER10* ), proteins involved in acyl reduction (*ASAT1 and WSD1*) and the decarbonylation pathway (*CER1*, *CER2*, *CER3*) and protein invovlved in wax or cuticle biosynthesis (LACS3).

### A transcription factor involved in the cuticle biosynthesis pathway

*SHINE1* encodes a member of the ERF (ethylene response factor) subfamily B-6 of the ERF/AP2 transcription factor family. Aharoni *et al*. [15] reported that *Arabidopsis* plants overexpressing *SHINE1* had a greater cuticular wax load than wild-type plants, and that this feature was associated with changes in alkane content conferring drought stress tolerance. Similar results have also been obtained for rice [32]. In the present study, a positive relationship was also found between *SHINE1* expression and CW content during the first growing season. Unfortunately, the lack of difference observed during the second growing season makes it difficult to speculate about the role of SHINE1 in CW biosynthesis. However, cuticular wax deposition is strongly regulated both developmentally and environmentally and ERF domains are known to be involved in the regulation of biotic and abiotic stress responses (reviewed by [32]). Further analyses of *SHINE1* expression in controlled environments are therefore required to determine whether this gene is involved in the regulation of CW deposition.

### VLCFA biosynthesis

**Figure 5:**
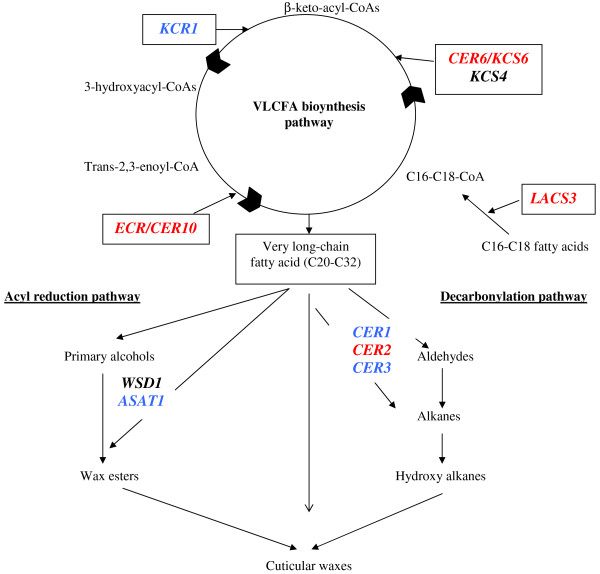
**Schematic representation of the metabolic pathway involved in cuticular wax biosynthesis.** A significant effect over the two growing seasons was observed for genes in red. For genes in blue, a significant effect was found only in 2008, and genes in black displayed very little variation of expression.

Needle waxes consist mostly of aliphatic compounds with 29 carbon atoms (Additional file 1: Figure S1). The VLCFA biosynthesis pathway is therefore a crucial element of CW biosynthesis in maritime pine. *CER6*/*KCS6*, *KCS4*, *KCR1* and *ECR*/*CER10* encode subunits of the acyl-CoA elongase complex, which is directly involved in VLCFA biosynthesis. *CER6*/*KCS6* and *KCS4* encode ketoacyl-CoA synthases, whereas *KCR1* and *ECR*/*CER10* encode a ketoacyl-CoA reductase and an enoyl-CoA reductase, respectively (Figure 5). By contrast, LACS3 encodes a long-chain acyl-CoA synthetase that is probably responsible for producing C16-C18-acyl-CoA from the C16-C18 fatty acids synthesized *de novo* in the plastids [6]. Its role may therefore be indirect (Figure 5). Consistent with their functions in cuticle biosynthesis, most of these genes were found to be upregulated in epidermal cells of *Arabidopsis thaliana* and in the drought-resistant *Arabidopsis myb 96*-*1D* activation mutant [3,33]. In the present study, these genes were overexpressed in the “V+” family plants in conditions of drought stress in early summer, but only *CER6*/*KCS6* and *ECR*/*CER10* displayed significant levels of overexpression in both growing seasons (T and/or TG effects, Figure 4). T, G, and TG effects were detected for *KCR1* in 2008, but this gene was slightly, but not significantly upregulated in early summer 2009.

### Acyl reduction pathway

GC analyses showed that primary alcohols accounted for 10% of the total wax load, whereas wax esters were not detected (Additional file 1: Figure S1), suggesting that the acyl reduction pathway was not the major wax biosynthesis pathway in maritime pine needles. In *Arabidopsis thaliana*, *WSD1* encodes the wax synthase [34] involved in the final reaction of the acyl reduction pathway (Figure 5). Costaglioli *et al*. [5] reported that *ASAT1* (At3g51970) may also encode a wax synthase, whereas Bouvier-Navé *et al*. [35] reported a possible role for this gene in sterol homeostasis. Shu *et al*. [33] reported the overexpression of this gene in stem epidermis, but no study has investigated the regulation of the *ASAT1* gene with respect to CW content and/or composition. In the present study, *ASAT1* was significantly downregulated in early summer 2008, with the highest levels of expression observed for plants of the “V+” subjected to the non-irrigated treatment. However, as we detected no wax esters in maritime pine needle waxes, this result must be interpreted with caution.

### Decarbonylation pathway

Alkanes and their derivatives (alkenes and secondary alcohols) accounted for more than 70% of the total wax load (nonacosan-10-ol alone accounting for 55 to 60%) (Additional file 1: Figure S1). Thus, as in Arabidopsis, the decarbonylation pathway is the main route of wax biosynthesis in maritime pine needles. The precise biochemical function of the CER1, 2 and 3 proteins in *Arabidopsis* remains unclear, but an analysis of the CW of the corresponding *cer* mutants (*cer1*, *cer2* and *cer3*) revealed major changes in alkane content, providing strong evidence of a role for the products of these genes in the decarbonylation pathway (reviewed by [2]). The upregulation of these three genes (*CER1*, *CER2*, *CER3*) in the non-irrigated plants of the “V+” family should be considered in light of the phenotypic results. Except for needles harvested on 7 July 2008, higher CW contents (significant treatment effect) were observed for the non-irrigated treatment (Figure 3). Kim *et al*. [36] and Cameron *et al*. [12] also reported a higher CW content in soybean and tobacco exposed to drought stress. As shown by the changes in CW composition over the two growing seasons (Additional file 4: Figure S3), C28 and C30 fatty alcohols were the only primary alcohols for which significant treatment effects were detected, whereas such effects were detected for the monounsaturated alkanes C29 hydroxy-alkene, C31 alkene and hydroxy alkene at most of the sampling times analyzed (Additional file 4: Figure S3). Kosma *et al*. [13] reported that cuticle thickness was greater in *Arabidopsis thaliana* subjected to water deficit than in well watered plants, and this greater cuticle thickness was essentially associated with large increases in the alkane constituents of the cuticle. Similarly, *CER1* overexpression in *Arabidopsis* resulted in a higher alkane content and greater drought stress tolerance [14]. Thus, these studies strongly suggest changes in cuticle composition and epicuticular wax content, in particular, are an adaptive response of the plant to water deficit. In this study, the higher CW content of non-irrigated plants of the “V+” family (in late summer) was associated principally with an increase in levels of monounsaturated alkanes.

## Conclusions

The upregulation of genes involved in VLCFA biosynthesis (CER6/KCS6, KCS4, KCR1, ECR/CER10 and potentially LACS3) and decarbonylation (CER1, CER2, CER3) may therefore have resulted in higher levels of alkanes and their derivatives in the cuticles of plants subjected to the non-irrigated treatment. Overall, these results suggest that such cuticle modification may allow family “V+” plants to cope more effectively with drought stress conditions, suggesting a possible role for the cuticle of maritime pine in drought stress adaptation. This hypothesis is supported by the higher growth rate (i.e. total height, Table 1 and Figure 1) of family “V+” plants subjected to the non-irrigated treatment.

This study provides also promising expressional candidate genes for future forward genetics studies. They will serve as a stepping stone to further work on drought stress adaptation in conifers for which few data are currently available.

## Methods

### Plant material

Two maritime pine half-sib families with known, different, adult growth rates were used to investigate the relationship between cuticular wax-associated gene expression and growth performance. The improved family (called “V+” for vigor +) was obtained by crossing an elite mother with high breeding value for height growth at eight years (1301–5,) with 30 males in a polycross design. All these trees were taken from the second generation of the maritime pine breeding program. The non-improved family (“V-” for vigor -) was obtained by a controlled cross between a mother tree “3802” (with low breeding value) and 30 other males, again in a polycross design. All these trees were taken from the first generation of the maritime pine breeding program [37,38]. Seeds were sown in turf plugs in May 2007 and were stored at the INRA nursery until February 2008, before plantation. The experiment was followed for two growing seasons (2008 and 2009).

### Drought stress

Drought stress was applied by excluding rainfall, by growing saplings in a 9 m wide and 58 m long greenhouse. The sides and ends of the greenhouse were opened to a height of 1 m (sides) and 2 m (ends), to mimic the external environmental conditions as closely as possible. Saplings were assigned to one of two areas: (i) irrigated or (ii) non-irrigated over two growing seasons. Overhead sprinkler irrigation was used to provide 10 mm of water three times per week (i.e. 30 mm per week), from April to October, in the irrigated zone. Ten rain gauges were also used to check that irrigation was uniform. Saplings from the “V+” and “V-” families were planted alternately, with a 40 x 40 cm square spacing, to ensure that they experienced the same water deficit or watering conditions. A 2 m buffer zone was left on either side of the greenhouse. An overview of the experimental design is provided in Additional file 5: Figure S4. Three samples were taken in each of the two growing seasons studied: in early spring (7 April 2008 and 8 April 2009), early summer (7 July 2008 and 27 July 2009) and late summer (22 September 2008 and 21 September 2009). Each sample was collected at 2 pm on a sunny day, to limit variation due to the circadian cycle. Before each sampling, stem length and collar diameter were measured for all saplings and used for the selection of 10 representative trees per family and set of modality. Needles were harvested independently from the same saplings for transcriptome and cuticular wax analyses. Needles were sampled on the last growth unit (i.e. the growth unit formed between two sampling points). The needles used for expression studies were immediately frozen in liquid nitrogen and stored at −80°C until RNA extraction, whereas the needles used for cuticular wax quantification were subjected to direct extraction in chloroform (see below).

### RNA extraction and real time quantitative PCR (qPCR)

Total RNA was extracted as described previously [39]. For each sampling point (family and treatment), total RNA was extracted from three independent biological replicates. One biological replicate was obtained by bulking needles harvested from three individuals. qPCR analyses were then carried out as described by Paiva *et al*. [40]. Reactions were carried out in a final volume of 20 μl, on a Chromo4™ Multicolor Real-Time PCR Detection System (Bio-Rad Laboratories, Inc. Hercules, CA, USA). Data were analyzed with Genex macro (Gene Expression Analysis for iCycle iQ® Real-time PCR Detection System, v1.10, 2004, Bio-Rad Laboratories), which uses a method based on the algorithms of Vandesompele *et al*. [41]. Before analysis, data were normalized against steady-state expression levels for both a gene encoding a 40S ribosomal protein (GE066D02, accession BX252550) and a gene encoding an unknown protein (RN44F10, accession BX677784). A full description of the primers used is provided in Additional file 2: Table S1. Statistical analyses were also performed, as previousely described by [42] and [43] to validate the expression profile obtained.

### Predawn leaf water potential

On each sampling date, leaf water potential was measured before dawn, with a Scholander-type pressure chamber. Measurements were made for six trees in each genetic unit, for both treatments, following standard procedure [44].

### Cuticular waxes analysis

Cuticular waxes (CW) were extracted and analyzed, as described by Bourdenx *et al*. [14]. CW were extracted from 500 mg of needles immersed for 90 s in chloroform containing docosane (1 mg/ml) as an internal standard. CW were quantified in three biological replicates for each sampling point, family and treatment. Biological replicates were generated as for RNA extraction. CW were quantified for needles harvested on 7 July 2008, 22 September 2008, 8 April 2009, 27 July 2009 and 21 September 2009.

### Estimation of water content

Water content of the same biological replicates used for CW analysis was determinated to show that the water content of the needles in all the experimental plot was not changed. Briefly, for each biological replicate 500 mg of fresh needles were dried for 3 days in a drying oven at 65°C. Water content was then estimated as follow: WC = ((Fresh Weight-Dry Weight/Fresh Weight)*100).

### Statistical analysis

The following linear model: Y_ijk_ = μ + T_i_ + G_j_ + (G*T)_ij_ + ϵ_ijk_ , where T_i_ is the treatment effect (i = irrigated vs. non-irrigated), G_j_ the family effect (j = “V+” *vs*. “V-”) and (T_i_*G_j_) is the interaction effect, was used, with *P*-values < 0.05 considered to identify significant discriminating points.

## Competing interests

The authors declare that they have no competing interests.

## Authors’ contributions

GLP and FD analyzed the data and co-wrote the manuscript with DB, CP, CL and PRC. AG carried out the qPCR analysis. DB, CM and FD were involved in the management of the experimental design. FD performed CW analysis. JMG coordinated the project. All the authors read and approved the manuscript.

## Supplementary Material

Additional file 1: Figure S1 Gas chromatogram of the cuticular waxes extracted from *Pinus pinaster* needles. Cuticular waxes were extracted by chloroform dipping and hydroxyl groups were silylated before GC separation and FID analysis. Numbers correspond to unidentified compounds that were systematically present in the various samples analyzed. Click here for file

Additional file 2: Table S1 List of the primer pairs used for qPCR analysis. Abbreviations: Tm: annealing temperature, NA: not available. TC IDs were retrieved from the pine gene index available from the following URL: http://compbio.dfci.harvard.edu/tgi/cgi-bin/tgi/gimain.pl?gudb=pine. Click here for file

Additional file 3: Figure S2 Expression profile of cuticle biosynthesis genes over the two growing seasons (2008 and 2009). Expression profiles for 2008 are on the left, whereas those for 2009 are on the right. Squares and red lines are used for the “V+” family for the non-irrigated treatment and triangles and orange lines are used for the “V-” family for the non-irrigated treatment. Squares and dark blue lines are used for the “V+” family for the irrigated treatment and triangles and light blue lines are used for the “V-” family for the irrigated treatment. Error bars represent the standard deviation (*N* = 3). Results of two-way ANOVA are also indicated above each sampling point. Abbreviations are as follows: T: treatment effect, G: family effect, TG: interaction effect. * *P* value < 0.01, **P value < 0.001 and ***P value < 0.0001. Click here for file

Additional file 4: Figure S3 Changes in cuticular wax composition over the two growing seasons. Red and orange bars correspond to the “V+” and “V-” families, respectively, for the non-irrigated treatment, whereas dark and light blue are used for the“V+” and “V-” families, respectively, for the irrigated treatment. Standard deviations were obtained from 3 measurements. Abbreviations correspond to: T: treatment effect, G: family effect, TG: interaction effect. * *P* value < 0.01. Unknown compounds were excluded from ANOVA. Click here for file

Additional file 5: Figure S4 Overview of the trial. **Panel A**: Global view of the greenhouse. The non-irrigated area is in the foreground of the picture. **Panel B**: Illustration of the sprinkler irrigation system. **Panel C**: Gutter to drain off rainwater, **Panel D**: Hobo logger to monitor environmental conditions, **Panel E**: soil moisture sensor, **Panel F**: piezometer to measure water-table level. Click here for file
