# Supplementary material for: Soil water stress affects both cuticular wax content and cuticle-related gene expression in young saplings of maritime pine (Pinus pinaster Ait)
Source: BMC Plant Biol. 2013 Jul 1;13:95. doi: 10.1186/1471-2229-13-95 (PMC3728238; doi:10.1186/1471-2229-13-95)
Supplement: Additional file 1: Figure S1 — Gas chromatogram of the cuticular waxes extracted from Pinus pinaster needles. Cuticular waxes were extracted by chloroform dipping and hydroxyl groups were silylated before GC separation and FID analysis. Numbers correspond to unidentified compounds that were systematically present in the various samples analyzed. [file 1471-2229-13-95-S1.doc]

**Additional File1-Figure S1: Gas chromatogram of the cuticular waxes extracted from *Pinus pinaster* needles**. Cuticular waxes were extracted by chloroform dipping and hydroxyl groups were sialylated before GC separation and FID analysis. Numbers correspond to unidentified compounds that were systematically present in the various samples analyzed.

15

15.5

16

16.5

17

17.5

18

18.5

19

0

20000

40000

60000

80000

100000

120000

140000

**Retention Time (min)**

**FID Response (counts)**

**1**

**2**

**3**

**4 5**

**C24-OH**

**C26-OH**

**C29AlkN**

**C28-OH**

**C31AlkN**

**C29AlkN-OH**

**C30-OH**

**C29-10OH**
