# Supplementary material for: Soil water stress affects both cuticular wax content and cuticle-related gene expression in young saplings of maritime pine (Pinus pinaster Ait)
Source: BMC Plant Biol. 2013 Jul 1;13:95. doi: 10.1186/1471-2229-13-95 (PMC3728238; doi:10.1186/1471-2229-13-95)
Supplement: Additional file 2: Table S1 — List of the primer pairs used for qPCR analysis. Abbreviations: Tm: annealing temperature, NA: not available. TC IDs were retrieved from the pine gene index available from the following URL: http://compbio.dfci.harvard.edu/tgi/cgi-bin/tgi/gimain.pl?gudb=pine. [file 1471-2229-13-95-S2.pdf]

Supplementary Table 1: List of the primer pairs used for qPCR. Abbreviations: Tm: annealing temperature, NA: not available. TC IDs were retrieved from the pine gene index available from the following URL: http://compbio.dfci.harvard.edu/tgi/cgi-bin/tgi/gimain.pl?gudb=pine.

Gene ID	Metabollic pathway	TC ID from TIGR[a]	*Arabidopsis thaliana* accession number	Function	Forward primer(5'-3')<br>Reverse primer (3'-5')	Amplicon size (bp)	Multiband in agarose gel	PCR efficiency (%)	Used in qPCR
KCS1	Fatty acid elongation	TC97038	At1g01120	3-ketoacyl-CoA synthase 1	CACCTTCATTGTCTGCGATG<br>TCCCTGCACTACAACCCATT	93	yes	NA	no
CER6/KCS6	Fatty acid elongation	CT581730	At1g68530	3-ketoacyl-CoA synthase 6	GGGTATGGCAGATTGCATTT<br>CAGTCCAGCCATGGATTCTT	103	no	94	**yes**
CER10/ECR	Fatty acid elongation	TC81572	At3g55360	enoyl-CoA reductase	GGGAAACCAGTTGTTCTGGA<br>CTGTGGCCCTAAATCCTTGA	99	no	110	**yes**
KCS4	Fatty acid elongation	BX675144	At1g19440	3-ketoacyl-CoA synthase4	CGTCGGTGAGCAAAGTACAA<br>AGCTTGCCAGCTTCATCATT	107	no	103	**yes**
KCR1	Fatty acid elongation	TC81537	At1g67730	beta-ketoacyl reductase1	ACCTGGGAAGAATCTCAGCA<br>TCTTGCGACGAGCTAACTGA	101	no	99	**yes**
FAR4	Cuticle biosynthesis	TC106034	At3g44540	fatty acyl-CoA reductases4	TTCTTGGCCGATCCAGATAC<br>CACAGTTTCCTGCGTGTTTG	97	yes	NA	no
FAR6	Cuticle biosynthesis	CT581080	At3g56700	fatty acyl-CoA reductases6	GCAATGGGAGAAATGCTAA<br>CGGGGAATGGATCAGAGTAA	101	yes	NA	no
LACS3	Cuticle biosynthesis	TC94979	At1g64400	long-chain acyl-CoA synthetase 3	GGTTTGAAGCAGGATGAAGC<br>GCTCCAGAAAGGATCAGACG	101	no	98	**yes**
ASAT1	Wax biosynthesis	TC97664	At3g51970	acyl-CoA sterol acyl transferase 1	CAGCAGGCAAATTCAGAACA<br>AAAACTGGGGGAAAACGAAT	104	no	104	**yes**
CER1	Wax biosynthesis	TC98775	At1g02205	unknown protein	CGAGGGCACTCGTTTTATTC<br>TTTTGGGCACACTCATTGAA	98	no	114	**yes**
CER2	Wax biosynthesis	TC96332	At3g23840	unknown protein	AGTTGTGATCAAGGGGATGC<br>TGTGATCTCCTCCCTGAACC	100	no	102	**yes**
CER3	Wax biosynthesis	TC83548	At5g02310	unknown protein	AATTGCAGTTCGACGGTTTC<br>CAGTGCTGCATTTGCCTTTA	103	no	102	**yes**
WSD1	Wax biosynthesis	TC108140	At5g53390	O-acyltransferase (WSD1-like) family protein	GTGGGGTTTTGAAATGGATG<br>CTTGGACGAAATTCCGATTG	102	no	102	**yes**
WSD2	Wax biosynthesis	TC85121	At5g55340	membrane bound O-acyl transferase	TTTGGCATGTAAACGGTCAA<br>TGCCATTGTCAAGAAGGCTA	100	yes	NA	no
WSD3	Wax biosynthesis	TC85022	At5g55380	membrane bound O-acyl transferase	ATGTGATAATGGGCGTGGTT<br>CGCCGTTAGTCTCAGCTTTT	101	yes	NA	no
SHINE1	Regulation of cuticle biosynthesis	TC98718	At1g15360	ERF/AP2 transcription factor	CTTCCCATTCAATCCCAATG<br>CCTTGAATCGAGGCCAGATA	102	no	101	**yes**
CYP96	Cutin	TC91954	At4g32170	Cytochrome P450 familly 96	GCTTCTGGGGATGATGTGTT	100	yes	NA	no

	biosynthesis				CCCAGATGCTTTCCATCCTA				
Lp3-3	Drought stress responsive genes	TC171388	NA	ABA inducible genes	GCTCACAGGCACAAGATTGA<br>CCTCCGCTTCCTTTTCTTCT	112	no	97	**yes**
PrAGP4	Drought stress responsive genes	TC182033	NA	Arabinogalactan protein 4	GAATGGCTATGGGAAACGAA<br>CTCTCGCTTGAGCCTTAGGA	122	no	99	**yes**
