# Supplementary material for: Soil water stress affects both cuticular wax content and cuticle-related gene expression in young saplings of maritime pine (Pinus pinaster Ait)
Source: BMC Plant Biol. 2013 Jul 1;13:95. doi: 10.1186/1471-2229-13-95 (PMC3728238; doi:10.1186/1471-2229-13-95)
Supplement: Additional file 3: Figure S2 — Expression profile of cuticle biosynthesis genes over the two growing seasons (2008 and 2009). Expression profiles for 2008 are on the left, whereas those for 2009 are on the right. Squares and red lines are used for the “V+” family for the non-irrigated treatment and triangles and orange lines are used for the “V-” family for the non-irrigated treatment. Squares and dark blue lines are used for the “V+” family for the irrigated treatment and triangles and light blue lines are used for the “V-” family for the irrigated treatment. Error bars represent the standard deviation (N = 3). Results of two-way ANOVA are also indicated above each sampling point. Abbreviations are as follows: T: treatment effect, G: family effect, TG: interaction effect. * P value < 0.01, **P value < 0.001 and ***P value < 0.0001. [file 1471-2229-13-95-S3.doc]

**Additional File 3- Figure S2: Expression profiles of cuticle biosynthesis genes over the two growing seasons (2008 and 2009).** Expression profiles for 2008 are on the left, whereas those for 2009 are on the right. Squares and red lines are used for the “V+” family for the non-irrigated treatment and triangles and orange lines are used for the “V-” family for the non-irrigated treatment. Squares and dark blue lines are used for the “V+” family for the irrigated treatment and triangles and light blue lines are used for the “V-” family for the irrigated treatment. Error bars represent the standard deviation (*N*=3). Results of two-way ANOVA are also indicated above each sampling point. Abbreviations are as follows: T: treatment effect, G: family effect, TG: interaction effect. * *P* value<0.01, ***P* value<0.001 and ****P* value<0.0001.

***Cuticle biosynthesis genes***

**T*****

**G*****

**TG****

**T***

**2008**

**T***

**2009**

**T***

**2008**

*KCS4*

**NA**

**G***

**T****

**G****

**TG*****

**2008**

**2009**

**T*****

**G*****

**TG*****

**T***

**2008**

**NA**

**2008**

**2009**

**T***

**TG***

**T***

**TG***

**T****

**G****

**TG***

**T*****

**G*****

**TG*****

**2008**

**2009**

**TG***

**T*****

**G*****

**TG*****

**T***

**G****

**TG***

**2008**

**TG***

**2009**

***Control genes involved in drought stress response n maritime pine***

**T****

**2008**

**T****

**2009**

**T***

**T***

**2009**

**T****

**T***

**T***

**NA**
