# Supplementary material for: Soil water stress affects both cuticular wax content and cuticle-related gene expression in young saplings of maritime pine (Pinus pinaster Ait)
Source: BMC Plant Biol. 2013 Jul 1;13:95. doi: 10.1186/1471-2229-13-95 (PMC3728238; doi:10.1186/1471-2229-13-95)
Supplement: Additional file 4: Figure S3 — Changes in cuticular wax composition over the two growing seasons. Red and orange bars correspond to the “V+” and “V-” families, respectively, for the non-irrigated treatment, whereas dark and light blue are used for the“V+” and “V-” families, respectively, for the irrigated treatment. Standard deviations were obtained from 3 measurements. Abbreviations correspond to: T: treatment effect, G: family effect, TG: interaction effect. * P value < 0.01. Unknown compounds were excluded from ANOVA. [file 1471-2229-13-95-S4.doc]

**Additional File4-Figure S3**: Changes in cuticular wax composition over the two growing seasons. Red and orange bars correspond to the“V+” and “V-” families, respectively, for the non-irrigated treatment, whereas dark and light blue are used for the“V+” and “V-” families, respectively, for the irrigated treatment. Standard deviations were obtained from 3 measurements. Abbreviations correspond to: T: treatment effect, G: family effect, TG: interaction effect. * *P* value<0.01. Unknown compounds were excluded from ANOVA

Total unknown

**7 July 2008**

24OH 26OH 28OH 30OH Alkene C29 AlkeneC31 C29 Hydroxy Alkene

10-hydroxy-C29 secondary alcohol

10-hydroxy-C29 secondary alcohol

Total unknown

**T***

**T***

**T***

**22 September 2008**

**T***

24OH 26OH 28OH 30OH Alkene C29 AlkeneC31 C29 Hydroxy Alkene

**T***

**T***

**T***

**T***

**T***

**T***

24OH 26OH 28OH 30OH Alkene C29 AlkeneC31 C29 Hydroxy Alkene

10-hydroxy-C29 secondary alcohol

Total unknown

**8 april 2009**

**T***

**T***

**T***

**T***

**T***

**27 July 2009**

10-hydroxy-C29 secondary alcohol

Total unknown

24OH 26OH 28OH 30OH Alkene C29 AlkeneC31 C29 Hydroxy Alkene

24OH 26OH 28OH 30OH Alkene C29 AlkeneC31 C29 Hydroxy Alkene

**21 September 2009**

**T***

**T***

**T***

10-hydroxy-C29 secondary alcohol

Total unknown
