# Supplementary material for: Soil water stress affects both cuticular wax content and cuticle-related gene expression in young saplings of maritime pine (Pinus pinaster Ait)
Source: BMC Plant Biol. 2013 Jul 1;13:95. doi: 10.1186/1471-2229-13-95 (PMC3728238; doi:10.1186/1471-2229-13-95)

Additional File 5-Figure S4: Overview of the trial. Panel A: Global view of the greenhouse. The non-irrigated area is in the foreground of the picture. Panel B: Illustration of the sprinkler irrigation system. Panel C: Gutter to drain off rainwater, Panel D: Hobo logger to monitor environmental conditions, Panel E: soil moisture sensor, Panel F: piezometer to measure water-table level.

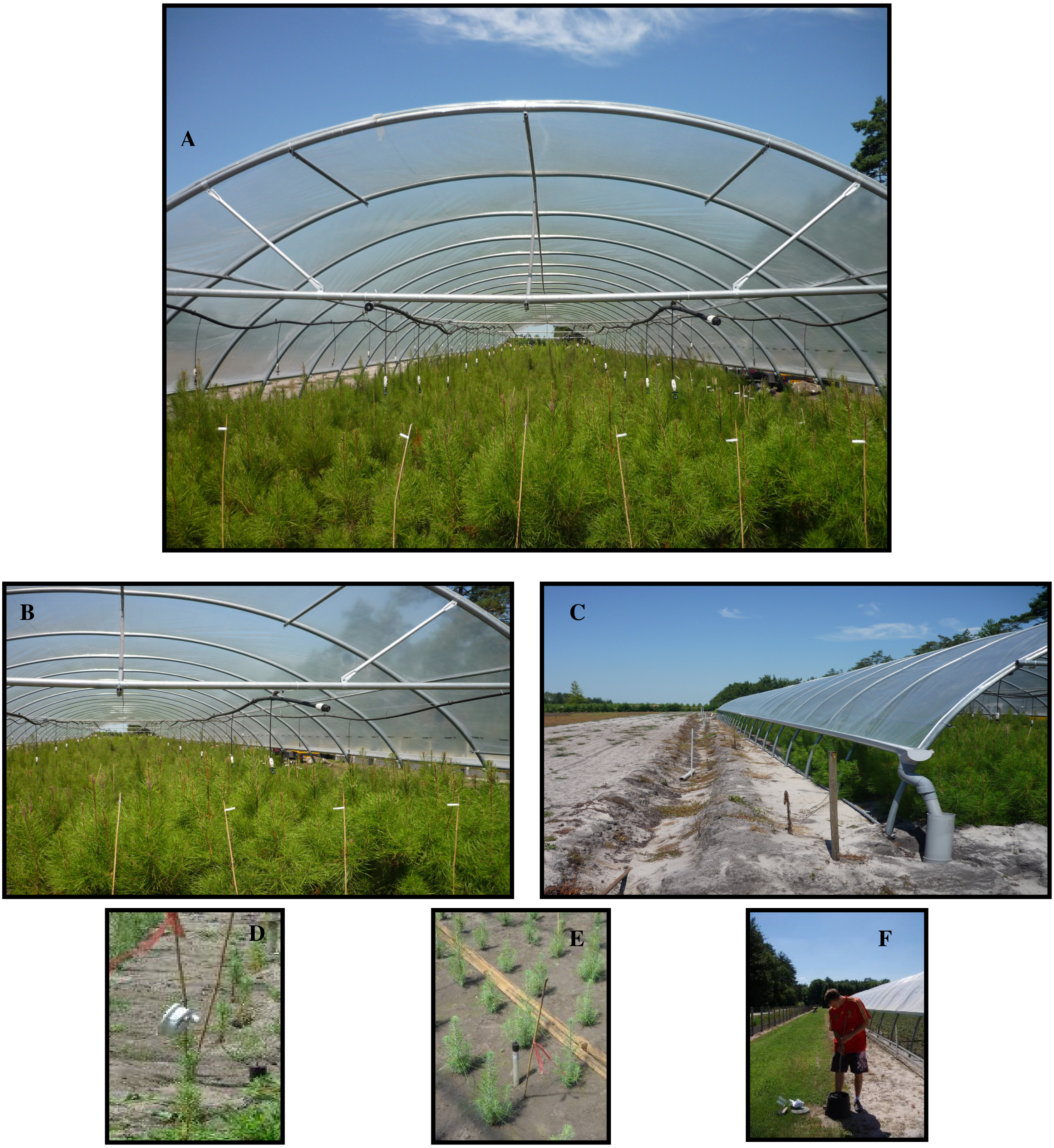

Supplement: Additional file 5: Figure S4 — Overview of the trial. Panel A: Global view of the greenhouse. The non-irrigated area is in the foreground of the picture. Panel B: Illustration of the sprinkler irrigation system. Panel C: Gutter to drain off rainwater, Panel D: Hobo logger to monitor environmental conditions, Panel E: soil moisture sensor, Panel F: piezometer to measure water-table level. [file 1471-2229-13-95-S5.pdf]
